# Meningiomas: An Overview of the Landscape of Copy Number Alterations in Samples from an Admixed Population

**DOI:** 10.1155/2020/3821695

**Published:** 2020-06-29

**Authors:** Michele Amaral da Silveira, Wallax Augusto Silva Ferreira, Carolina Koury Nassar Amorim, José Reginaldo Nascimento Brito, André Salim Kayath, Fernanda do Espirito Santo Sagica, Edivaldo Herculano Corrêa de Oliveira

**Affiliations:** ^1^Programa de Pós-Graduação em Neurociências e Biologia Celular, ICB, UFPA, Rua Augusto Correa, 01, Belém, PA 66075-990, Brazil; ^2^Laboratório de Cultura de Tecidos e Citogenética, Seção de Meio Ambiente, Instituto Evandro Chagas, BR 316 Km 7, s/n Levilândia, Ananindeua, PA, Brazil; ^3^Programa de Pós-Graduação em Oncologia e Ciências Médicas, NPO, Universidade Federal do Pará (UFPA), Rua dos Mundurucus 4487, Belém, PA, Brazil; ^4^Núcleo de Pesquisas Oncológicas, Universidade Federal do Pará (UFPA), Rua dos Mundurucus 4487, Belém, PA, Brazil; ^5^Faculdade de Ciências Exatas e Naturais, ICEN, Universidade Federal do Pará, Rua Augusto Correa, 01, Belém, PA 66075-990, Brazil

## Abstract

Meningiomas are considered the most common intracranial tumors, affecting mainly women. Studies in mixed populations can be of great importance to clarify issues related to the genetic diversity of tumors and their development. Considering that data obtained from analyses of the profile of copy number alterations (CNA) have been a useful diagnostic indicator for many types of tumors and that meningiomas show a complex pattern of gains and losses in the number of copies, our objective was to analyze the CNA profile in 33 samples of meningiomas of different histological grades (WHO Grade I-III) from patients in a city located in the Amazon region of Brazil, using aCGH. We found that the female to male ratio was 3 : 1. The aCGH analysis revealed a total of 2304 CNA, with an average of 69.8 ± 57.4 per case, of which 1197 were gains (52%), 926 were losses (40.2%), 105 were amplifications (4. 5%), and 76 were deletions (3.3%). A significant relationship was observed between the type of CNA and the degree of the tumor (chi-square test: *χ*^2^ = 65,844; *p* < 0.0001; contingency coefficient: *C* = 0.1772; *p* < 0.0001). Evaluating the recurrent changes in at least 50% of the samples, we observe as the most frequent losses of the segments 22q13.1-q13.2 (82%), 1p35.3 (76%), and 14q13.1-q13.2 (67%), involving all histopathological grades. The analysis of these regions showed the inclusion of genes with functions such as regulation, maintenance of cell survival, reorganization of the cytoskeleton, cell signaling, and DNA repair, among others. However, overall, the profiles observed in meningiomas of this admixed population were very similar to the ones observed in Caucasian groups. An interesting finding was a recurrent gain of 8p22 observed only in grade I meningiomas, a region which includes DLC1, a suppressor candidate gene probably implicated in the developments or progression of meningiomas, usually found deleted, when related to CNAs.

## 1. Introduction

Meningiomas are among the most common of central nervous system (CNS) neoplasms, corresponding to more than a third of primary CNS neoplasms and being considered the most common intracranial tumors [1–3]. They arise from meningothelial arachnoid cells and are often attached to the internal surface of the dura mater [4, 5]. They are mostly benign and slow growing, although a small proportion present malignant behavior, characterized by invasive growth patterns and/or significantly higher recurrence rates [6]. Meningiomas are more common in adults, accounting for 38% of intracranial tumors in women and 20% in men [7]. The incidence of meningiomas increases markedly after the age of 65 and with the aging of the population, and studies on this tumor are becoming increasingly prevalent in neuro-oncology [3].

The World Health Organization (WHO) classification categorizes meningiomas based on their histological characteristics and risk of recurrence, in three grades: I, benign (80%), II, atypical (18%), and III, anaplastic/malignant (2%). Studies suggest that the aggressive behavior of some meningiomas is attributed to molecular changes, regardless of their histopathology, and histological aspects of each variant may indicate specific biomolecular changes [2, 8, 9]. Thus, it is opportune to understand and integrate the genomic findings that have the greatest impact in determining the clinical and biological behavior of meningiomas, aiming to incorporate them in the future, in the pathological classification of these tumors.

In the past few years, our understanding of meningioma biology, classification, grading, and molecular genetics have enabled the identification of recurring genetic and epigenetic alterations that are promising treatment targets [2, 10]. In this sense, the analyses of the profile of copy number alterations (CNA) have shown that meningiomas are characterized by a complex pattern of gains and losses of segments harboring not only tumor suppressor genes and oncogenes but also candidate genes or genes with an already known role in tumorigenesis pathways [11, 12]. Several candidate genes are associated with tumorigenesis in meningiomas, with a complex pattern of gains and losses in the number of copies throughout the genome [13].

For instance, the most common CNAs in meningiomas involve chromosomes 22q, which are found in the neurofibromatosis 2 (*NF2*) gene. Allelic losses (loss of heterozygosity (LOH)) of this region are found in 40–70% of sporadic and the vast majority of NF2 associated meningiomas. Additionally, NF2 mutations are found in up to 60% of tumors, consistent with a classic two-hit mechanism of tumor suppressor gene inactivation [2, 14–17]. Usually, it is proposed that meningiomas progress from low-grade to high-grade tumors, although this is not always easy to be demonstrated [18], and cytogenetic studies propose a higher amount of chromosomal gains and losses according to the grade of tumors [19–24]. Whence, while LOH at 22q12.2 represented an early event, gains or amplifications involving 1q, 9q, 12q, 15q, 17q, and 20q have been associated with higher grade [25–27].

An important aspect to consider is that it is becoming clear that some of the differences in cancer risk, incidence, and survival among people of different racial and ethnic backgrounds can be attributed to biological factors [28]. Hence, studies in admixed populations may have a great importance to clarify issues concerning the genetic diversity of tumors and its development, considering that most studies have been performed in Caucasians.

In this regard, Northern Brazilian populations are extremely interesting, because they are derived from the intermixture of Amerindians, Europeans, and Africans [28–30]. Moreover, Carvalho et al. [31] identified that meningiomas represented the second most frequent CNS tumor type in a public cancer hospital in the State of Pará, analyzing samples between 1997 and 2014. Based on this, our objective was to analyze the landscape of copy number alterations in samples of meningiomas from patients in a city located in the Amazon region of Brazil, considering that other studies involving non-mixed populations may not be applied to this. The results may show whether genomic alterations are common among Caucasian and miscegenated populations, or if they represent a different pattern in the latter, in view of the lack of this type of study in non-Caucasian populations.

## 2. Material and Methods

### 2.1. Patients

Thirty-three fresh tumor biopsies were evaluated, comprising primary meningioma tumors belonging to the three WHO malignancy grades, and collected between 2014 and 2018. Clinical data for each patient were obtained, and the summary of the characteristics is shown in Table 1. The present study was approved by the Ophir Loyola Hospital Ethical Committee (ID 593.717-0).

### 2.2. DNA Isolation

The DNA from biopsies was isolated using Illustra tissue and cells genomic Prep Mini Spin kit (GE Healthcare), according to the protocols provided by the supplier. The 260/280 and 260/230 ratios were determined by NanoDrop (Thermo Scientific) and the DNA was quantified using TapeStation (Agilent), using the supplier's protocol.

### 2.3. Array Comparative Genomic Hybridization (aCGH)

Array comparative genomic hybridization (aCGH) using SurePrint G3 CGH + SNP Array 180K platform (Agilent Technologies, CA, USA) was performed according to the Agilent Technologies protocol (Agilent Oligonucleotide Array-Based CGH for Genomic DNA Analysis Enzymatic Labeling for Blood, Cells, or Tissues, protocol v. 7.2, published in July 2012). In brief, 1 *μ*g of reference DNA (Agilent Euro Male/Female) and patient DNA was digested and labeled using the SureTag DNA Labeling kit (Agilent Technologies). After purification, labeled sample and reference DNA were cohybridized at 65°C for 16 hours to the array and washed and according to the supplier's default protocol. The slides were scanned and decoded by the software Feature Extraction v. 10.7 (Agilent Technologies), using the protocol CGH_107_Sep09.

### 2.4. Data Analyses

Data were visualized and analyzed by Agilent Cytogenomics 5.0 as described elsewhere [32]. Aberration statistical algorithm ADM-2, with threshold 6.0, was used for CNAs. Five-probe 0.15_log2 filter was used for aberration evaluation. For analyses, we considered CNAs found in two or more samples, which were organized using the software Excel (Microsoft). For the evaluation of gains or losses in the number of copies of DNA segments, only those that covered at least five consecutive oligonucleotides with log2 of the test/reference ratio ≥0.3 or ≤−0.3 were considered as possible changes in the number of copies, gains and losses, respectively.

The proportions between the sexes were calculated using Fisher's exact test, considering *p* < 0.05. The mean, median, standard deviation, and amplitude for the age variable were calculated. The comparison of the means between the ages by sex was performed using the Student's *t*-test. Pearson's correlation analysis was performed to verify whether there was a relationship between age and tumor grade according to the WHO. The chi-square test and the correlation test (verifying the association between variables) “contingency coefficient *C*,” which is a nonparametric model that verifies the presence of associations between qualitative ordinal variables, were performed. The tests were used to verify whether there is a relationship between the type of CNA and the degree of the tumor according to the WHO. All statistical analyses were performed using the GraphPad Prism 8.0.2 (GraphPad Software; La Jolla, CA) software.

## 3. Results

### 3.1. Samples

**Figure 1:**
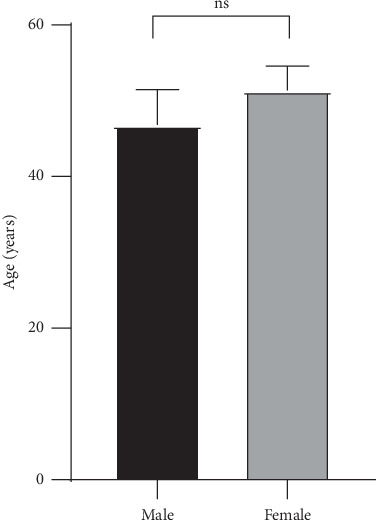
Average age for sex. Bars: mean with SEM.

We analyzed 33 fresh samples of meningiomas, of which twenty-one patients were females (64%) and twelve patients were males (36%) (Table 1). The female to male ratio was 3 : 1, according to Fisher's exact test (*p* < 0.05). The ages ranged from 17 to 84 years, and the age of the MNG 105 case was not known. The total mean age was 49.8 ± 14.9 (Table 2). Student's *t*-test was used to compare the mean age according to gender, with the male gender being 46.8 and the female gender was 51.3, with *p*=0, 20, demonstrating that there was no significant difference between them (Figure 1).

Of the thirty-three samples, twenty-six were classified as benign (grade I), two as grade II, and one sample as grade III. Four did not present information regarding the histological type (Table 3). Of those classified as grade I, nine were meningothelial, five were fibroblastic, four were transitional, two were psammomatous, three were mixed (with characteristics of two histological types), one was syncytial, one was microcytic, and one was hemangioblastic. Those classified as grade II were clear cell meningiomas and the one classified as grade III was anaplastic meningioma. There was no association between age and grade of the tumor (*p*=0.2976).

### 3.2. Copy Number Alterations

aCGH analysis revealed a total of 2304 CNAs in the 33 samples of meningiomas evaluated, with an average of 69.8 ± 57.4 per case (ranging from 27 to 264), of which 1197 were gains (52%), 926 were losses (40.2%), 105 were amplifications (4.5%), and 76 were deletions (3.3%).

Table 4 shows the total number of CNAs according to histological grades, in which the gains were allocated with the amplifications, while losses were allocated with the deletions. It was found that grade I samples showed 60.3% gains/amplifications, grade II samples showed 79.6% gains/amplifications, and grade III samples showed 73% losses/deletions.

**Figure 2:**
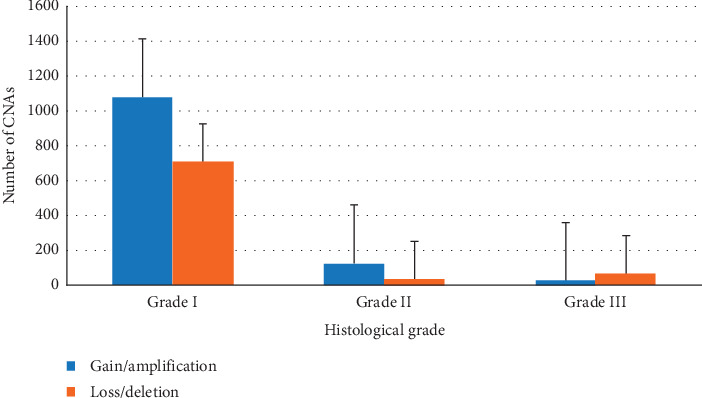
Type of CNA in relation to histological grade.

A significant relationship was observed between the type of CNA and the degree of the tumor (chi-square test: *χ*^2^ = 65,844; *p* < 0.0001; contingency coefficient: *C* = 0.1772; *p* < 0.0001). Grade III sample showed more CNAs of losses/deletions when compared to grades I and II, which presented more gains/amplifications (Figure 2).

Taking into account the recurrent CNAs in at least 50% of the studied samples, it was possible to identify a total of 85 CNAs, involving chromosomes 1, 3, 4, 8, 11, 14, 15, 16, 17, and 22. The most frequent CNAs were loss/deletion in 1p (1p35.3–76%), 3q (3q29–54%), 4p (4p14–57%), 11q (11q23.3–51%), 14q (14q13.1-q13.2–67%), 15q (15q15.1–57%), 16q (16q22.1–54%), and 22q (22q13.1-q13.2–82%) and gain/amplification in 8p (8p.22–64%), 14q (14q32.33–100%), 16q (16q.21–51%), and 17q (17q21.33–67%).

The analysis of these regions showed the inclusion of genes with functions such as regulation, maintenance of cell survival, reorganization of the cytoskeleton, cell signaling, and DNA repair, among others, which are associated with the appearance of several types of pathologies.  Table 5 highlights some of these genes found in these regions, correlated with the histological grade.

## 4. Discussion

We have profiled genomic CNAs of primary meningioma tumors belonging to the three WHO malignancy grades, with a total of 33 samples, in order to compare our results obtained from a miscegenated, genetically distinct population with other previous studies, which mostly include Caucasian populations. Overall, our results were very similar to other studies, not only concerning the characterization of the sample, such as sex ratio, age, and subtypes, but also considering the copy number alteration profile.

In what concern the general aspects of the samples, we found a 3 : 1 ratio between females and males, showing an average age of 49.8, similar to the findings by Mendes et al. [33], who found an average of 47 years and the highest number of women affected in a study conducted in Southern Brazil. Carvalho et al. [31], who analyzed the same population, also reported a greater number of women in the group of patients with meningiomas. Approximately 80% of the cases of meningiomas described in the literature are benign and correspond to grade I according to the current WHO classification, with the meningothelial subtype being the most frequent, followed by fibroblastic and transitional, which combines the two patterns mentioned above, with transitions between both [34]. Holleczek et al. [4] found in their study the proportion of 70%, 28%, and 3% of meningiomas of grades I, II, and III, respectively, according to the WHO classification.

The population studied in the present study was mostly composed of grade I type and meningothelial subtype, followed by fibroblast, and these findings are similar to those found in other populations in relation to the frequency of the type of histological grade, despite the fact of being ethnically quite distinct of the population analyzed by those authors. There was no association between age and grade of the tumor. Sporadic meningiomas are characterized by several changes in the number of chromosomal copies, and it seems to increase with the degree and progression of the tumor [35–38]. We found a total of 2304 CNAs in the 33 samples of meningiomas evaluated, of which 1197 were gains (52%), 926 were losses (40.2%), 105 were amplifications (4.5%), and 76 were deletions (3.3%).

A significant relationship was observed between the type of CNA and the degree of the tumor, indicating that grade III presented more loss/deletion when compared to grades I and II. According to previous reports, atypical and anaplastic meningiomas include frequent loss of chromosomes 1p, 6q, 10q, 14q, and 18q and gain of chromosomes 1q, 9q, 12q, 15q, 17q, and 20q. Among these, the most frequent changes observed are losses in 1p and 14q, apart from alterations in chromosome 22q which correspond to the most frequently observed ones in meningiomas, usually affecting the *NF2* gene, and are present in half of grade II and almost all grade III meningiomas [6, 10, 20]. In the present study, taking into account the analyses carried out on CNAs present in at least 50% of the samples, we observed that the losses/deletions involved chromosomes 1p, 3q, 4p, 11q, 14q, 15q, 16q, and 22q and the gains/amplifications involved chromosomes 8p, 14q, 16q, and 17q. Most changes were found on chromosomes 22, 1, and 14, respectively, encompassing samples of all histopathological grades.

Among characterized genetic alterations, loss of an entire chromosome 22 is commonly reported in meningiomas and was among the first recurring cytogenetic alterations ever described in human solid tumors, even when meningiomas were studied by means of classical cytogenetics [39]. The importance of CNAs involving this chromosome remains evident, as they are detected in virtually all studies involving genomic analyses of meningiomas.

Concerning losses/deletions in 1p, the most frequently identified one was 1p35.3 (76%), a region where we find several important genes, including *SESN2* that encodes a protein that can act in the regulation of cell growth and survival and may still be involved in the cellular response to different stress conditions [40, 41]. This CNA was found in samples of all histopathological grades and may be associated with the development and progression of meningiomas. In addition to this, it is worth noting the importance of molecular profile in the identification of subgroups of meningiomas. Hence, loss of *RCC1* is associated with the development of microcystic meningioma, and this alteration was observed in one of our samples, classified as microcystic, a type which corresponds to 1% of all grade I meningiomas.

Most recurrent losses are related to regions containing genes related to tumor suppression pathways, while gains usually include potential oncogenes. Additionally, most alterations found in grade I tumors were also observed in grades II and III, suggesting they may be related to the process of initial development of meningiomas. The only recurrent alteration exclusive for grade I tumor was the gain of 8p22. Interestingly, this region is usually related to loss, as it harbors *DLC1*, a suppressor candidate gene probably implicated in the developments or progression of meningiomas [42–45]. Hence, the correct sense of this amplification in a high proportion of grade I meningiomas remains unclear.

Overall, our results are similar to the landscape of copy number alterations described in other populations that are ethnically different, suggesting that meningiomas are not influenced by genetic variability related to ethnicities. In fact, the similarity of our findings with previous reports may be indicative of the relative importance of some of these CNAs as specific driver alterations, especially the ones identified markedly different across grades.

## Figures and Tables

**Table 1:** Patient information regarding sex, age, and histological classification of meningiomas.

Sample ID	Sex	Age (years)	Histological type	Grade
MNG 02	Male	25	Transitional meningioma (mixed)	I
MNG 06	Male	41	Malignant meningioma (anaplastic)	III
MNG 16	Female	61	Fibroblastic meningioma with low cellularity and no mitotic activity	I
MNG 23	Male	48	Meningioma (clear cell type areas)	II
MNG 26	Female	55	Transitional meningioma	I
MNG 32	Male	56	Mixed meningioma	I
MNG 37	Female	37	Clear cell meningioma	II
MNG 38	Male	31	Meningothelial meningioma	I
MNG 44	Female	46	Meningothelial meningioma	I
MNG 45	Female	46	Transitional meningioma	I
MNG 46	Female	48	Fibroblastic meningioma	I
MNG 54	Female	74	Fibroblastic meningioma	I
MNG 57	Male	41	Atypical meningothelial meningioma	I
MNG 66	Female	63	Fibroblastic meningioma	I
MNG 75	Male	46	Syncytial meningioma	I
MNG 77	Male	84	Transitional meningioma	I
MNG 78	Female	65	Psammomatous meningioma	I
MNG 81	Female	59	Meningothelial meningioma	I
MNG 83	Female	63	Psammomatous meningioma	I
MNG 84	Male	54	Transitional meningioma	I
MNG 98	Female	75	Microcystic meningioma	I
MNG 105	Male	—	Meningioma^*∗*^	
MNG 112	Male	49	Meningothelial meningioma	I
MNG 119	Female	61	Meningioma^*∗*^	
MNG 122	Female	55	Fibroblastic meningioma	I
MNG 123	Female	32	Meningioma^*∗*^	
MNG 125	Female	57	Mixed meningioma	I
MNG 33	Female	40	Hemangioblastic meningioma with cellularity of the forehead and mitotic index	I
MNG 45	Male	40	Meningothelial meningioma	I
MNG 46	Female	17	Meningothelial meningioma	I
MNG 50	Female	51	Meningothelial meningioma	I
MNG 142	Female	45	Meningothelial meningioma	I
MNG 201	Female	29	Meningioma^*∗*^	

^*∗*^Histological type was not reported. —, age was not informed.

**Table 2:** Descriptive statistics of patient ages.

	Age
Maximum	84
Minimum	17
Mean	49.8
Median	48.5
SD	14.8
Amplitude	67
Total	32 samples

**Table 3:** Information on patients regarding the degree of the tumor.

Grade	*N*
I	26
II	2
III	1
Not categorized	4

**Table 4:** Total number of CNAs by histological grade.

	Gains/amplifications	Losses/deletions	Total number of CNAs
Grade I	1078	707	1785
Grade II	125	32	157
Grade III	24	65	89

**Table 5:** Some genes found in the most frequent altered cytobands in the analyzed meningioma samples correlating the histological grade.

Cytobands	Genes	Histological grade	Type of CNA^*∗*^	Frequency in the samples (%)
1p35.3	*THEMIS2*	I, II, III	Loss	76
	*RPA2*			
	*SESN2*			
	*RCC1*			
3q29	*PAK2*	I, III	Loss	54
4p14	*RFC1*	I, III	Loss	57
8p22	*MSR1*	I	Gain	64
11q23.3	*CXCR5*	I, III	Loss	51
14q13.1-q13.2	*EAPP*	I, II, III	Loss	67
14q32.33	*FAM30A*	I, II, III	Gain	100
15q15.1	*CHAC1*	I, II, III	Loss	57
16q21	*CDH11*	I, II	Gain	51
16q22.1	*SF3B3*	I, III	Loss	54
17q21.33	*COL1A1*	I, II	Gain	67
22q13.1-q13.2	*ST13*	I, II, III	Loss	82
	*TOB2*			

^*∗*^Consider loss/deletion and gain/amplification.

## Data Availability

The complete results from the aCGH analyses, used to support the findings of this study, are included within the supplementary information file.

## Supplementary Materials

Supplementary Materials Data sheet containing all the detected CNAs with respective chromosomal location, detected by aCGH in the multisample analyses.
